# Neutralization of Omicron BA.4/BA.5 and BA.2.75 by booster vaccination or BA.2 breakthrough infection sera

**DOI:** 10.1038/s41421-022-00472-5

**Published:** 2022-10-13

**Authors:** Xun Wang, Jingwen Ai, Xiangnan Li, Xiaoyu Zhao, Jing Wu, Haocheng Zhang, Xing He, Chaoyue Zhao, Rui Qiao, Minghui Li, Yuchen Cui, Zixin Hu, Chenqi Xu, Wenhong Zhang, Pengfei Wang

**Affiliations:** 1 State Key Laboratory of Genetic Engineering, Shanghai Institute of Infectious Disease and Biosecurity, School of Life Sciences, Fudan University, Shanghai, China; 2 Department of Infectious Diseases, Shanghai Key Laboratory of Infectious Diseases and Biosafety Emergency Response, National Medical Center for Infectious Diseases, Huashan Hospital, Fudan University, Shanghai, China; 3 State Key Laboratory of Molecular Engineering of Polymers, Department of Macromolecular Science, Fudan University, Shanghai, China; 4 Institute of Biochemistry and Cell Biology, Shanghai Institutes for Biological Sciences, Chinese Academy of Sciences, Shanghai, China; 5 Artificial Intelligence Innovation and Incubation Institute, Fudan University, Shanghai, China; 6 State Key Laboratory of Genetic Engineering, Collaborative Innovation Center for Genetics and Development, School of Life Sciences and Human Phenome Institute, Zhangjiang Fudan International Innovation Center, Fudan University, Shanghai, China; 7 National Clinical Research Center for Aging and Medicine, Huashan Hospital, Fudan University, Shanghai, China

**Keywords:** Molecular biology, Immunology

Dear Editor,

With the continued mutation of severe acute respiratory syndrome coronavirus 2 (SARS-CoV-2) Omicron variant, many new Omicron sub-lineages have been reported to evade neutralizing antibodies induced by both vaccination and infection, including BA.2, BA.2.12.1, BA.4 and BA.5^1–3^. Most recently, another emerging sub-lineage BA.2.75^4^, carrying nine additional mutations in spike compared to BA.2 (Fig. 1a), has been reported in multiple countries. Our previous study showed that homologous or heterologous booster can remarkably reduce Omicron BA.1, BA.1.1, BA.2 and BA.3 escape from neutralizing antibodies^5^, but a comprehensive neutralization assessment of booster vaccination or breakthrough infection sera against all the distinct emerging Omicron sub-lineages is still lacking.

**Fig. 1:**
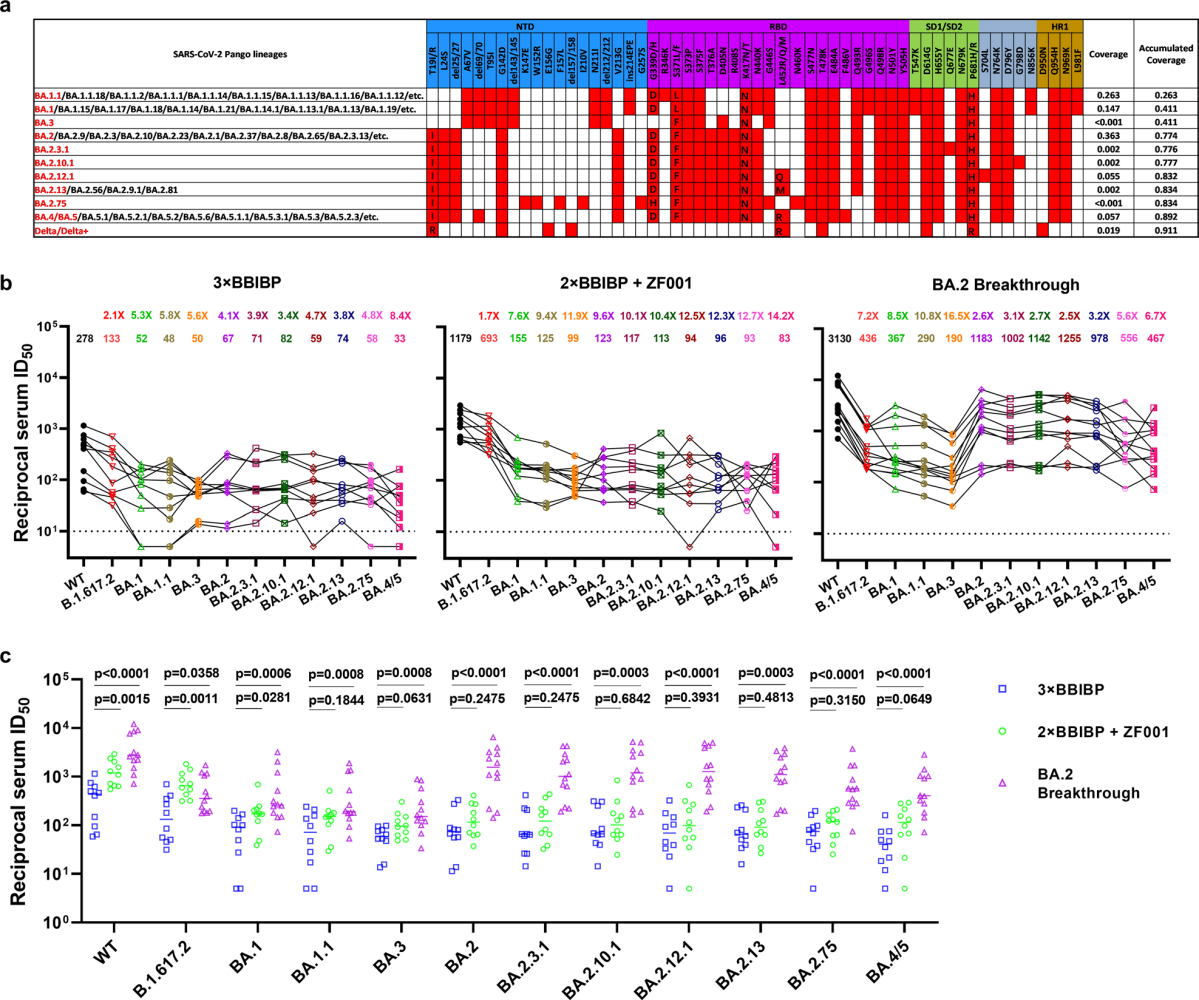
Characteristics and sera neutralization of the Omicron sub-lineages. **a** Prevalence and spike mutations of the Omicron sub-lineages and Delta variant based on all the sequences available in GISAID since Jan 1st, 2022. Coverage indicates the total percentage of the indicated lineages in GISAID database. **b** Neutralization of pseudotyped WT (D614G), Delta and Omicron sub-lineage viruses by sera collected from individuals on day 14 after vaccination with a BBIBP-CorV homologous booster or with a ZF001 heterologous booster dose following two doses of BBIBP-CorV, or infection by BA.2 virus after three doses of BBIBP-CorV vaccination. For all panels, values above the symbols denote GMT and the fold-change was calculated by comparing the titer to WT. **c** In parallel comparison of neutralization titers against distinct SARS-CoV-2 variants by sera collected from individuals on day 14 after vaccination with homologous or heterologous booster, or breakthrough infection with BA.2 virus. *P* values were determined by multiple Mann-Whitney tests.

Here, apart from the four Omicron sub-lineage pseudoviruses (PsVs) that we already had from our previous study, we further constructed a panel of PsVs based on BA.2, including BA.2.3.1, BA.2.10.1, BA.2.12.1, BA.2.13, BA.2.75 and BA.4/BA.5. Some of these Omicron sub-lineages bear identical spike protein with many other sub-lineages evolving from BA.1, BA.2 or BA.5, and thus our virus panel can represent many more Omicron sub-lineages regarding their neutralization evasion levels (Fig. 1a). We also included Delta (B.1.617.2) variant in this study due to its recent replacement by Omicron. Taken all these variants and their representing variants into account, their accumulated coverage reached 91% according to the proportion of sequences deposited in GISAID database since Jan 1st, 2022 (Fig. 1a), which is the most comprehensive panel of Omicron sub-lineages tested as we know.

We collected serum samples from healthy adults on day 14 post homologous booster with BBIBP-CorV, or heterologous booster with ZF2001, primed with two doses of BBIBP-CorV (Supplementary Table S1), and tested their neutralization activity on this panel of PsVs. This study was approved by the ethical committee of Huashan Hospital Affiliated to Fudan University (number KY2022-596 & KY2021-749). The written informed consents had been obtained from all the enrolled patients. As shown in Fig. 1b and Supplementary Fig. S1, the homologous booster group (3× BBIBP, *n* = 10) displayed a neutralizing geometric mean titer (GMT) of 278 against wild-type (WT, D614G), with 2.1- to 8.4-fold reduction against Delta and Omicron sub-lineages. For the heterologous booster group (2× BBIBP + ZF2001, *n* = 10), this cohort had higher neutralizing titers with GMTs of 1179, 693, 155, 125, 99, 123, 117, 113, 94, 96, 93 and 83 against WT, Delta, BA.1, BA.1.1, BA.3, BA.2, BA.2.3.1, BA.2.10.1, BA.2.12.1, BA.2.13, BA.2.75 and BA.4/5, respectively. Although these numbers amount to 1.7- to 14.2-fold reductions in potency for the Delta and Omicron sub-lineages compared to WT, nearly all samples retained detectable neutralizing activity against these distinct variants. Compared with BA.4/5, BA.2.75 obtains additional mutations in the N-terminal domain but lacks two critical mutations (L452R and F486V) in the receptor-binding domain (RBD). Therefore, although BA.2.75 accumulated the largest number of mutations in its spike, BA.4/5 showed the strongest serum escape in both the homologous and heterologous booster groups.

The SARS-CoV-2 BA.2 variant has led to an increasing number of breakthrough infections in China. To gain further insight into their chance of re-infection by new Omicron sub-lineages, we recruited 12 convalescents immunized with three-dose inactivated vaccines prior to infection with Omicron BA.2 and evaluated their serum samples on day 14 post-infection on the same panel of PsVs. We found that BA.2 breakthrough infection significantly increased neutralizing antibody to higher titers with GMTs of 3130, 436, 367, 290, 190, 1183, 1002, 1142, 1255, 978, 556 and 467 against WT, B.1.617.2, BA.1, BA.1.1, BA.3, BA.2, BA.2.3.1, BA.2.10.1, BA.2.12.1, BA.2.13, BA.2.75 and BA.4/5 (Fig. 1b). For BA.2, its derivative variants and BA.4/5, the reduction levels compared to WT in the breakthrough infection group were lower than those of the homologous and heterologous vaccine booster groups. While for Delta, BA.1, BA.1.1 and BA.3, the reduction levels were much higher, which may be associated with the antigenic difference between Omicron BA.2 and these other variants.

To further understand the differences between vaccination and BA.2 breakthrough infection, we compared in parallel the serum neutralization titers of homologous booster, heterologous booster, and BA.2 breakthrough infection, against different viruses. Heterologous booster exhibited higher titers than homologous booster against WT and Delta variant. However, the neutralization titers for Omicron sub-lineages showed no difference between homologous and heterologous (RBD-subunit) boosters, which could be attributed to the large number of mutations accumulated in Omicron variants, especially those in RBD. More importantly, we found that BA.2 breakthrough infection significantly increased neutralizing antibody titers to relatively high levels compared with homologous and heterologous booster vaccination against almost all variants (Fig. 1c). For the BA.2 breakthrough infection sera, we further compared the neutralization titers against BA.2 with those against the other variants. We observed significantly lower titers against BA.2.75 and BA.4/5, which harbor several additional mutations over BA.2, than that against BA.2; while for the other sub-lineages like BA.2.3.1, BA.2.10.1 and BA.2.12.1, which are derived from BA.2 with only one additional mutation, we observed similar response to the breakthrough infection sera compared to BA.2 (Supplementary Fig. S2).

Taken together, our results demonstrated that all Omicron sub-lineages showed substantial evasion of neutralizing antibodies induced by vaccination, with BA.4/5 being the most significant one. However, BA.2 breakthrough infection could remarkably elevate neutralization titers against all different variants, especially titers against BA.2 and its derivative sub-lineages.

## Supplementary information

Supplementary Materials
