# Supplementary material for: Neutralization of Omicron BA.4/BA.5 and BA.2.75 by booster vaccination or BA.2 breakthrough infection sera
Source: Cell Discov. 2022 Oct 13;8:110. doi: 10.1038/s41421-022-00472-5 (PMC9557992; doi:10.1038/s41421-022-00472-5)
Supplement: Supplementary file 1 — Supplementary Materials [file 41421_2022_472_MOESM1_ESM.pdf]

Supplementary Materials for:

Neutralization of Omicron BA.4/BA.5 and BA.2.75 by Booster Vaccination or BA.2 Breakthrough Infection Sera

Xun Wang [1,#], Jingwen Ai [2,#], Xiangnan Li[3#], Xiaoyu Zhao[1,#], Jing Wu[2,#], Haocheng Zhang[2,#], Xing He[4,#], Chaoyue Zhao[1], Rui Qiao[1], Minghui Li[1], Yuchen Cui[1], Zixin Hu[3,5,*], Chenqi Xu[4,*], Wenhong Zhang[2,6,*], Pengfei Wang[1,*]

[1]State Key Laboratory of Genetic Engineering, Shanghai Institute of Infectious Disease and Biosecurity, School of Life Sciences, Fudan University, Shanghai, China

[2]Department of Infectious Diseases, Shanghai Key Laboratory of Infectious Diseases and Biosafety Emergency Response, National Medical Center for Infectious Diseases, Huashan Hospital, Fudan University, Shanghai, China

[3]State Key Laboratory of Genetic Engineering, Collaborative Innovation Center for Genetics and Development, School of Life Sciences and Human Phenome Institute, Zhangjiang Fudan International Innovation Center, Fudan University, Shanghai, China

[4]Institute of Biochemistry and Cell Biology, Shanghai Institutes for Biological Sciences, Chinese Academy of Sciences, Shanghai, China

[5]Artificial Intelligence Innovation and Incubation Institute, Fudan University, Shanghai, China

[6]National Clinical Research Center for Aging and Medicine, Huashan Hospital, Fudan University, Shanghai, China

[#]These authors contributed equally

[*]Address correspondence to Pengfei Wang (pengfei_wang@fudan.edu.cn), Wenhong Zhang (zhangwenhong@fudan.edu.cn), Chenqi Xu (cqxu@sibcb.ac.cn) or Zixin Hu (huzixin@fudan.edu.cn).

Supplementary materials and methods

Serum samples

Sera from individuals who received three doses of BBIBP-CorV or two doses of BBIBP-CorV plus ZF2001 vaccine were collected at Huashan Hospital, Fudan University 14 days after the final dose. Sera were also obtained from patients after 14 days of SARS-CoV-2 breakthrough infection caused by Omicron BA.2 variant (confirmed by sequencing) after immunizing with three-dose inactivated vaccines (CoronaVac or BBIBP-CorV). All collections were conducted according to the guidelines of the Declaration of Helsinki and approved by the Institutional Review Board of the Ethics Committee of Huashan Hospital (2021-041 and 2021-749). All the participants provided written informed consents.

Construction and production of variant pseudoviruses

Plasmids encoding the WT (D614G) SARS-CoV-2 spike and Omicron sub-lineage spikes, as well as the spikes with single or combined mutations were synthesized. Expi293F cells were grown to 3×10^6/mL before transfection with the indicated spike gene using Polyethylenimine (Polyscience). Cells were cultured overnight at 37 °C with 8% CO_2 and VSV-G pseudo-typed ΔG-luciferase (G*ΔG-luciferase, Kerafast) was used to infect the cells in DMEM at a multiplicity of infection of 5 for 4 h before washing the cells with 1×DPBS three times. The next day, the transfection supernatant was collected and clarified by centrifugation at 300g for 10 min. Each viral stock was then incubated with 20% I1 hybridoma (anti-VSV-G; ATCC, CRL-2700) supernatant for 1 h at 37 °C to neutralize the contaminating VSV-G pseudotyped ΔG-luciferase virus before measuring titers and making aliquots to be stored at −80 °C.

Pseudovirus neutralization assays

Neutralization assays were performed by incubating pseudoviruses with serial dilutions of monoclonal antibodies or sera, and scored by the reduction in luciferase gene expression. In brief, Vero E6 cells were seeded in a 96-well plate at a concentration of 2×10^4 cells per well. Pseudoviruses (1000 $TCID_{50}$ per well) were incubated the next day with serial dilutions of the test samples in triplicate for 30 min at 37 °C. The mixture was added to cultured cells and incubated for

an additional 24 h. The luminescence was measured by Luciferase Assay System (Beyotime). IC_{50} was defined as the dilution at which the relative light units were reduced by 50% compared with the virus control wells (virus + cells) after subtraction of the background in the control groups with cells only. The IC_{50} values were calculated using nonlinear regression in GraphPad Prism.

Analysis of spike mutations and prevalence of SARS-CoV-2 variants

The coverage of each lineage group was summarized by counting the submission records of each lineage in the same group since 2022 from GISAID database (Accessed on 30 July, 2022). The lineages were grouped together if they share same mutations in at least 60% submitted sequences in spike protein. Lineages with prefix "AY" were grouped as Delta+ lineage. The mutation data of each lineage were retrieved using R package outbreakinfo (https://github.com/outbreak-info/R-outbreak-info) with lookupSublineages() and getMutationsByLineage() functions. We then filtered and counted the submission records of each lineage and calculated the coverage of each lineage group. Only lineages with both mutation data and submission records were taken into account.

Supplementary Table S1. Baseline characteristics of enrolled participants, including Omicron BA.2 breakthrough infection, BBIBP-CorV homologous booster group and BBIBP-CorV/ ZF2001 heterologous booster group.

	Omicron BA.2 breakthrough infection (n=12)	BBIBP-CorV homologous booster dose (n=10)	BBIBP-CorV/ZF2001 heterologous booster dose (n=10)	P value
Age(years), median(range)	26(21-31)	28(19-33)	22.5(23-51)	0.02
Male, n(%)	4(33.33%)	4(40.00%)	3(30.00%)	0.90
BMI(kg/m^2),mean(SD)	22.02(2.52)	20.87(2.38)	21.18(2.4)	0.56
Comorbidities(%)				
Any, n(%)	1(8.30%)	1(10.00%)	0(0.00%)	0.63
Tumor, n(%)	0(0.00%)	1(10.00%)	0(0.00%)	
Others, n(%)	1(8.30%)	0(0.00%)	0(0.00%)	0.63

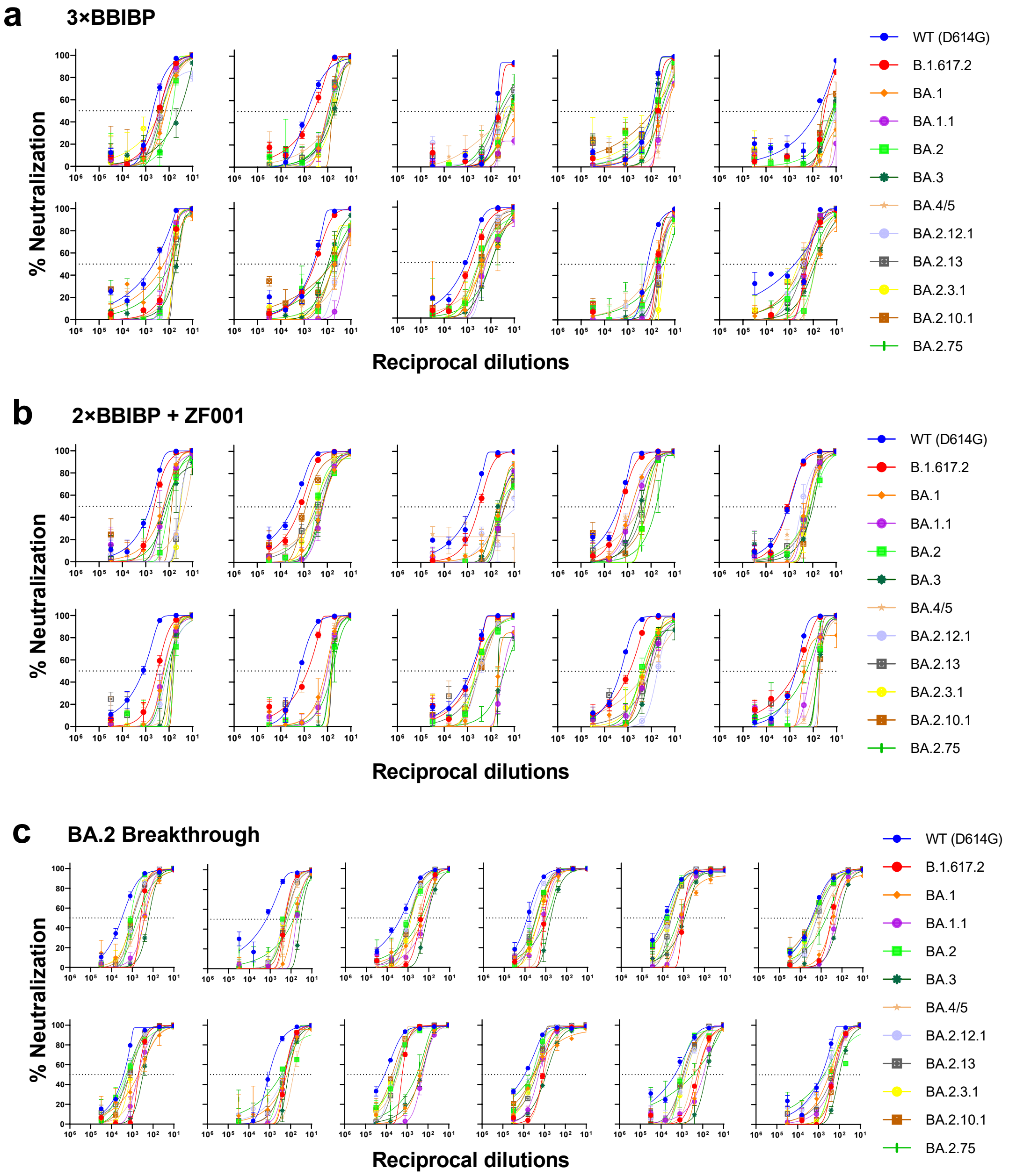

Supplementary Fig. S1: Neutralization curves for sera collected at day 14 post the BBIBP-CorV (**a**) or ZF001 booster dose (**b**) or BA.2 breakthrough infection (**c**)

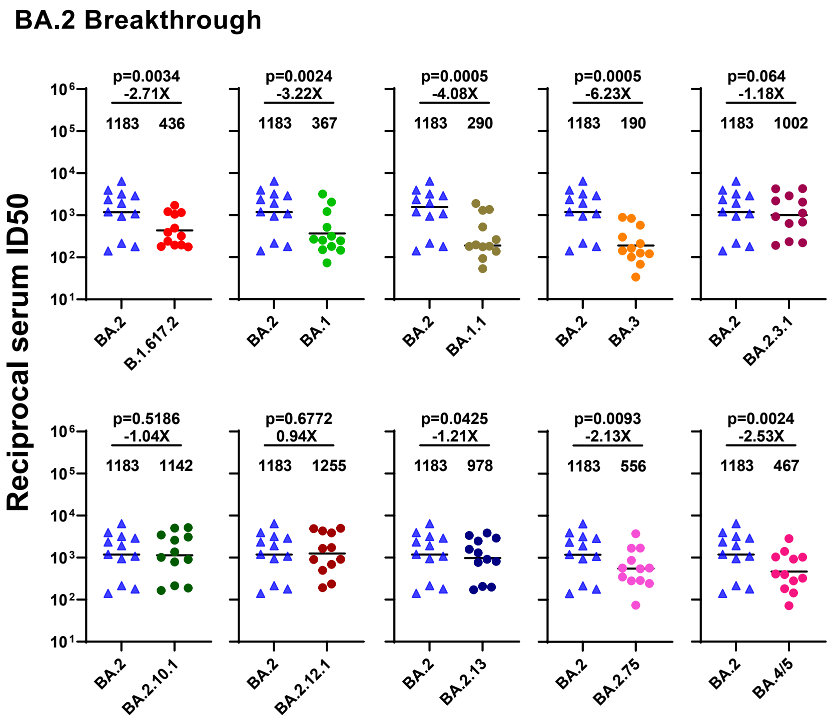

Supplementary Fig. S2: Neutralization activity comparison of BA.2 breakthrough infection sera against BA.2 and other variants.
